# Modeling the effects of a Staphylococcal Enterotoxin B (SEB) on the apoptosis pathway

**DOI:** 10.1186/1471-2180-6-48

**Published:** 2006-05-31

**Authors:** Brandon W Higgs, John Dileo, Wenling E Chang, Haley B Smith, Olivia J Peters, Rasha Hammamieh, Marti Jett, Jordan C Feidler

**Affiliations:** 1 Emerging Technologies Office, The MITRE Corporation, McLean, VA, USA; 2 Walter Reed Army Institute of Research (WRAIR), Silver Springs, MD USA

## Abstract

**Background:**

The lack of detailed understanding of the mechanism of action of many biowarfare agents poses an immediate challenge to biodefense efforts. Many potential bioweapons have been shown to affect the cellular pathways controlling apoptosis [1-4]. For example, pathogen-produced exotoxins such as Staphylococcal Enterotoxin B (SEB) and Anthrax Lethal Factor (LF) have been shown to disrupt the Fas-mediated apoptotic pathway [2,4]. To evaluate how these agents affect these pathways it is first necessary to understand the dynamics of a normally functioning apoptosis network. This can then serve as a baseline against which a pathogen perturbed system can be compared. Such comparisons can expose both the proteins most susceptible to alteration by the agent as well as the most critical reaction rates to better instill control on a biological network.

**Results:**

We explore this through the modeling and simulation of the Fas-mediated apoptotic pathway under normal and SEB influenced conditions. We stimulated human Jurkat cells with an anti-Fas antibody in the presence and absence of SEB and determined the relative levels of seven proteins involved in the core pathway at five time points following exposure. These levels were used to impute relative rate constants and build a quantitative model consisting of a series of ordinary differential equations (ODEs) that simulate the network under both normal and pathogen-influenced conditions. Experimental results show that cells exposed to SEB exhibit an increase in the rate of executioner caspase expression (and subsequently apoptosis) of 1 hour 43 minutes (± 14 minutes), as compared to cells undergoing normal cell death.

**Conclusion:**

Our model accurately reflects these results and reveals intervention points that can be altered to restore SEB-influenced system dynamics back to levels within the range of normal conditions.

## Background

Apoptosis is a naturally occurring mechanism by which a cell undergoes programmed death in response to external or internal signals. It has been shown that many potential biowarfare agents including Staphylococcal Enterotoxin B (SEB) improperly activate the pathways controlling this process in a variety of cell types [1-4]. These agents target specific components of these pathways, inducing a cascade of reactions that modify the rate at which apoptosis occurs. The large scale cell death that ensues brings about a state of unrecoverable shock.

Since the induction of apoptosis underlies the lethality of SEB, this pathway presents a good target for therapeutic intervention. Previous results have shown that SEB treatment affects the expression of proteins involved in the Fas-mediated apoptotic pathway [2,4]. However, the mechanisms by which these agents interact with this pathway, and the cellular components most advantageous for potential therapies, is unclear. In order to establish a better understanding of these interactions, it is first necessary to develop a model of the pathway as it functions under normal cellular conditions. This model can then serve as a baseline against which experimentally perturbed systems can be compared and key intermediates determined.

**Figure 1:**
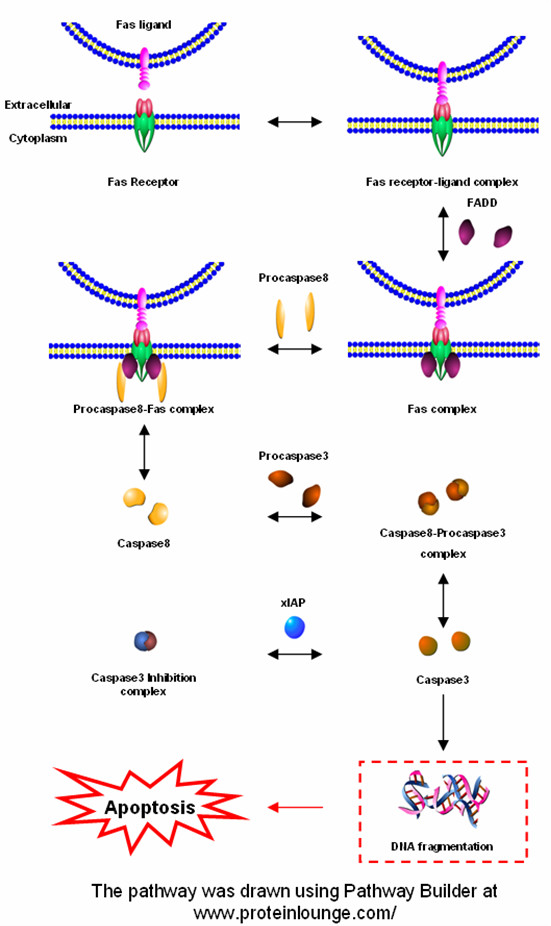
**Diagram of Fas-mediated apoptosis**. Model pathway representation of Fas-mediated apoptosis (modified from Fussenegger et al., 2000).

The model of receptor-mediated apoptosis is represented in Figure 1 and summarized as follows. The cascade begins with extracellular Fas ligand (FasL) binding to the cell surface receptor Fas/CD95, thus initializing aggregation and activation [5]. The adapter protein FADD binds to the Fas receptor through its death domain (DD) and transfers the apoptotic signal to the inactive forms of caspase-8 (procaspase-8) and caspase-3 (procaspse-3). This causes procaspase-8 to undergo a self proteolytic cleavage reaction resulting in its activation, as well as small traces of proteolytic cleavage of procaspase-3. Once activated, caspase-8 is instrumental in the subsequent activation of multiple other proteins [6]. Of particular interest is the subsequent proteolytic cleavage of procaspase-3 by caspase-8. Activation of caspase-3 rapidly results in DNA degradation and inactivation of other cellular functions [5]. The activation of executioner caspase-3 is also one of the final stages in cell death, thus providing an endpoint marker for verification of apoptosis.

A level of control over this process is provided by a family of proteins called inhibitors of apoptosis (IAPs). IAPs can intervene at the executioner caspase point to suppress cell death [7-9]. Leveraging this regulatory reaction can increase our understanding of the system dynamics to instil regulation points and control mechanisms.

Our main objectives within this exposition are two-fold. We developed a parsimonious model representation of Fas-mediated apoptosis under normal cellular conditions in an attempt to (1) reduce the complexity of the biological network to the most critical protein intermediates and (2) elucidate the most influential reaction rates for controlling an SEB-exposed system and reverting the system back to a normal cellular state. Our model [see Additional file 1] provides an accurate representation of the pathway dynamics, as evaluated by consistency with experimental data.

## Results

### Model of the normally functioning Fas pathway

In order to develop a model of Fas-mediated apoptosis under what we identify as 'normal' conditions, we stimulated human Jurkat cells with an anti-Fas antibody and determined the relative levels of seven proteins involved in the core pathway by western blot at 0, 2, 4, 8, and 12 hours after exposure. It should be noted that the introduction of a signal (the anti-Fas antibody in this case) for induction of apoptosis is artificial as compared to completely normal cellular conditions. However the primary comparison seeks to identify differences between non-pathogen induced and pathogen induced apoptosis. The measured protein levels were used to impute relative rate constants and build a quantitative model consisting of a series of ordinary differential equations (ODEs) that simulate the network under normal conditions. Flow cytometry was also conducted on the stimulated Jurkat cells at the time points specified to verify the presence of cell death and determine a difference in early apoptotic events between a normally functioning Fas pathway and one under the influence of SEB exposure (examined in the next section).

Under these conditions, the simulated results are in agreement with the experimental data. All proteins assayed, with exception to procaspase-8, correlate with data in our model with coefficient values of at least 0.70 (Table 1). From Figure 3a, the Fas receptor is shown to immediately decrease in level after activation with the anti-Fas activating antibody, while FADD increases with a strong inverse relationship (r = -0.72 between FADD and the Fas receptor). The simulated results from the inactive and active caspase-3 proteins reach successive peaks, with a time difference of 1.71 hours (Figure 3c), suggestive of the time required for proteolytic cleavage of the precursor form and subsequent activation. The mean protein levels from the experimental data follow the same pattern, with a time difference of 1.96 ± 1.63 hours between inactive and active caspase-3. This difference between average peaks in inactive and active caspase-3 for experimental and simulated results amounts to approximately 15 minutes. Such a difference is significantly small, when examined in the context of the biological reproducibility for each experimental curve. It is interesting to note the constitutive protein level of the X-linked inhibitor of apoptosis protein (xIAP) (Figure 3d). This protein has been shown previously to directly inhibit caspase-3 [10]. The regulation between xIAP and caspase-3 is examined in detail in the **Reaction rates space **section. The strong similarities between the experimental and simulated data indicate that this model describes this Fas-mediated mechanism.

The simulated procaspase-8 protein is least correlated with experimental results due to the abnormally large peak in level at ~ 4.5 hours, followed by a steep shift in slope direction after this point. Procaspase-8 is expressed at large protein levels, as compared to procaspase-3. Such a result is likely due to transcriptional activation of the procaspase-8 gene. Since this particular caspase is also involved in the internally-mediated (mitochondrial) mechanism of apoptosis, larger concentrations might be necessary for activation of competing protein cascades, not captured within this model representation. The simulated procaspase-8 curve resembles the experimental results up to the apex of the curve. After this point, the simulated curve continues to increase with positive slope, whereas the experimental curve decreased rapidly. The modeled representation of procaspase-8 was calculated to assume a protein expression pattern that both proceeds and resembles caspase-8, similar to the relationship exhibited between procaspase-3 and caspase-3. So this unexpected discrepancy between simulated and experimental results for procaspase-8 is likely the result of a transcriptional effect, not captured within the model representation.

### Model of the Fas pathway under the influence of SEB exposure

In order to develop a model of this pathway under SEB-influenced conditions, the previously described experiments were performed with the addition of recombinant SEB. Compared to normal conditions, the experimental data shows significant differences (using a threshold of p < 0.05) in protein level patterns for three of the seven critical proteins – procaspase-8, caspase-3, and xIAP. In addition, flow cytometry results for the SEB-exposed cells indicate a positive shift in density for annexin-stained cells, as compared to cells stimulated with only the anti-Fas antibody at ~ 8 hours (data not shown). This distribution difference in the former illustrates how exposure to SEB (and anti-Fas antibody) induces apoptosis at a greater rate than cells stimulated with the anti-Fas antibody. Table 2 contains the *T*^2 ^statistics, *F*-values, degrees of freedom (df), and p-values for the protein comparisons.

When visualizing the curve patterns for these three proteins, the differences between conditions are apparent (Figure 4). The inactive procaspase-8 protein reaches maximum levels at 4.41 ± 0.10 hours under normal cellular conditions (Figure 4a), whereas in SEB-exposed cells, this protein reaches maximum levels at 2.20 ± 0.22 hours (Figure 4b). This is a time lead difference of 2 hours 13 minutes (± 14 minutes).

The active caspase-3 protein shows a similar pattern to procaspase-8. Under normal cellular conditions, this protein reaches a maximum level at 4.65 ± 0.18 hours (Figure 4c) (approximately 14 minutes after procaspase-8), whereas under conditions of SEB exposure, caspase-3 reaches a maximum level at 2.94 ± 0.16 hours (Figure 4d). This is a time lead difference of 1 hour 43 minutes (± 14 minutes). The result that under SEB exposure, both caspase-3 and procaspase-8 show accelerated rates by approximately 2 hours implies that these two proteins are coupled to some mechanism induced by SEB. This association, however, is likely related to another pathway and not to this Fas-mediated model, since the intermediates in the cascade (procaspase-3 and active caspase-8) do not exhibit a similar accelerated pattern.

The xIAP protein seems to demonstrate the largest apparent difference of the three. Under normal cellular conditions, this protein is at a constitutive level (considering confidence bounds-Figure 4e), whereas under SEB-exposed conditions, this protein is down-regulated, or essentially degrading at a fast rate (Figure 4f). The maximum level for xIAP under SEB-exposed conditions is at *t *= 0. Previous work has demonstrated xIAP to be a direct inhibitor of caspase-3. Our experimental results support this finding in that under normal conditions, xIAP is at a constitutive level. However, under conditions of SEB exposure, xIAP is inhibited, or simply negatively affected, thereby reducing the repressive effect on caspase-3 and consequently increasing the rate of caspase-3. This result implies two notions: (1) SEB influences the degradation of xIAP, thereby both releasing a regulatory effect and increasing the rate of caspase-3, and subsequently, apoptosis, and (2) under SEB-exposed conditions, increasing the rate of xIAP can be a potential solution to restoring the level of caspase-3 and the normal rate of apoptosis. Such an implication is evaluated next for possible solutions within the rate constant space.

### Reaction rate space

To understand the conditions under which caspase-3 is both expressed normally and at an increased rate (under the influence of SEB exposure), the relative kinetic rate space is sampled to define regions of normal and perturbed cellular conditions. As was explained previously, caspase-3 is one of the final proteins leading to DNA damage and cell death in the Fas-mediated mechanism, so understanding the parameters under which this protein is synthesized provides control over the outcome of apoptosis. Such boundaries are necessary to identify those areas within the plane associated with the system behaviour. This perspective on the allowable rate space provides a regulatory mechanism to revert a perturbed cellular system back to a stable normal state.

Two possible scenarios were examined within this analysis. First, the two rate constants from the primary regulatory reaction between xIAP and caspase-3 were simulated for solutions under normal cellular conditions (Figure 1-ultimate reaction). This criterion encompasses normal caspase-3 synthesis, where the expression peak occurs at 4.65 hours and xIAP is either constitutively, or simply positively expressed (Figures 4c,e). The second scenario simulates the rate constants where the peak in expression of caspase-3 is increased from 4.65 hours and the expression of xIAP is a decreasing function (Figures 4d,f), resembling either degradation or decreasing expression. Possible example solutions to these two scenarios are provided in Figure 5. For each plot, the bold line represents the same average protein profile as those observed in Figure 4. The dashed lines represent examples of potential solutions that are simulated. Note that in the dashed line solutions, the protein levels that are simulated can vary, however, the peak rates and linear trends cannot.

Within the model, the primary regulatory reaction between xIAP and caspase-3 is dictated by two relative rate constants, allowing the plane to be readily visualized within two dimensions (Figure 6). This extraction of the solution space for the two rates under both conditions is represented with green circles for normal states and blue crosses for SEB-simulated states, where each point represents a rate value for the forward (k_15_) and reverse (k_16_) reaction. It is interesting to note the peak pattern for the possible rate values necessary to achieve SEB-exposed conditions (blue crosses). Such points are indicative of caspase-3 expression at an increased rate and a decreasing expression function for xIAP. As the k_16 _rate values increase, the range of k_15 _values begins to decrease, for allowable solutions to the SEB-exposed condition. Though the allowable rates for the model were restricted to the range of 1.0 × 10^-9 ^to 18 (justified in the **Methods **section), the y-axis (representative of k_16_) was simulated for values up to 70, to identify the highest point for the SEB-simulated solution space. The rate space under normal cellular conditions is restricted to two small areas within the plane: very small values of k_15 _and k_16_, and large values of k_16 _for k_15 _limited to the range of 5.5 to 9. Simulations using the rate values within these two small regions provide results associated with normal cellular conditions for caspase-3 and xIAP, as determined by agreement with experimental data. In attempt to revert a perturbed system from SEB-exposed to normal cellular conditions, this representation can guide the necessary directionality and magnitude of rate adjustments. For example, at relative rate values of k_15 _= 8 and k_16 _= 30, the system exhibits behaviour indicative of SEB exposure, however, an increase in the reverse rate constant (k_16_) to values greater than 40 can restore the expression of both caspase-3 and xIAP to behaviours simulating a normal system. This result is counterintuitive when first examining this reaction, mainly because an increase in the reverse reaction rate, k_16 _would imply an increase in the rate of caspase-3 synthesis, rather than a reduction. However, it is important to remember that k_16 _is the sole synthesis parameter for xIAP and the level of xIAP is but a single component of one degradation term for caspase-3. So k_16 _values that occur greater than a certain threshold (values greater than 40 in this example) dramatically increase xIAP to high levels, which simultaneously increases the degradation term of caspase-3, thus slowing down the rate. To simulate this result within a cellular system, this is analogous to increasing the cellular concentration of xIAP to a point where apoptosis can be slowed down, indicative of normal cellular conditions.

## Discussion

We have constructed a parsimonious model representation of Fas-mediated apoptosis under both normal and SEB-influenced cellular conditions. Our simulated results agree with experimental data as evaluated by correlation. In addition, experimental results demonstrate an increased rate of apoptosis and significant differences for three proteins (procaspase-8, caspase-3, and xIAP) between normal and SEB-exposed conditions.

In addition to the direct effect of SEB on the Fas pathway, there are several other factors that could lead to this acceleration of apoptosis. First, several factors could be causing an increase in FasL concentration. Specifically, both SEB treatment of various cell types (including Jurkats) and activation of the Fas receptor [13,14] have been shown to cause the *de novo *production and secretion of soluble FasL (sFasL). Also, SEB treatment has been shown to induce the production of several members of the (tumor necrosis factor) TNF superfamily such as TNFα and tumor necrosis factor-related apoptosis-inducing ligand (TRAIL) [11]. Increased TNFα levels have been shown to lead to proteolytic cleavage of existing FasL from the cell surface [12]. This increase in sFasL would create a positive feedback loop thus increasing the rate of apoptosis.

Second, the increase in TRAIL expression has been shown to initiate Jurkat cell apoptosis through the DR5 receptor [15]. In addition to promoting apoptosis, activation of this parallel pathway eventually leads to an increase in caspase-8 expression and may partially explain our results (discussed further below) [15]. The addition of other pathways into our minimal model will be the subject of further work.

Finally, the induction of fratricide apoptosis in which activated T cells begin inducing apoptosis in neighboring cell as a result of SEB exposure may play a role in this rate increase as well [16].

It is interesting to note that the increased rate for procaspase-8 is not complimented by an increase in proteolytic cleavage and subsequent activation of caspase-8. This implies that SEB is inducing the increased rate on the gene expression point, since the inactive caspase demonstrates a significant difference in the presence of SEB exposure, as opposed to the active caspase.

This accelerated rate is likely the result of SEB affecting several signal transduction pathways that regulate caspase-8 expression. Studies have shown that SEB stimulation of Jurkat cells causes them to begin secreting various cytokines such as interferon gamma (IFNγ) within 30 minutes following exposure [17-19] The release of IFN-γ can then increase the rate of transcription of caspase-8 in an interferon regulatory factor 1 (IRF1) dependent manner. [18,20,21].

The accelerated rate of caspase-3 and degradation of xIAP under SEB influence imply a repressive regulation of xIAP under normal conditions, such that SEB exposure releases the repressive effect and increases the rate of caspase-3 and subsequently, apoptosis. SEB has been shown to induce the activation of caspase-3 [12], however, the combined effect on the interaction (indirect or direct) between SEB and xIAP/caspase-3 is not well understood. Under the assumption of a mitochondrial mechanism (internally mediated apoptosis), SEB could inhibit xIAP similar to how synthetic Smac/DIABLO has been demonstrated to, thereby leading to an increased activity of caspase-3 and caspase-9 in etoposide-induced cells [22]. However, since autocrine release of fas was observed, and previous work has shown the SEB treatment induces FasL release [12], the mechanism is more likely not primarily mediated through the mitochondrial pathway, and instead, fas-mediated.

Simulation results also demonstrate a means to instil control on an SEB-exposed system. Utilizing the primary inhibitory reaction between caspase-3 and xIAP, the relative rate space was computed to illustrate values for converting a perturbed system back to normal cellular conditions. Such results indicate that at specific increased concentrations of xIAP, the rate of apoptosis can be reduced from an SEB-exposed rate to a normal cellular rate.

## Conclusion

In future work, we plan to expand the model to account for the protein intermediates that are observed to have an effect on procaspase-8, in addition to the indirect connection between increased rates of procapase-8 and caspase-3 under the influence of SEB.

## Methods

### Flow cytometry

After the cells became confluent in free RPMI1640 with 1.0 × 10^6^/ml cells, they were split equally into three flasks. One flask was incubated with anti-Fas (0.01 ug/ml) and the other was incubated with the combination of anti-Fas (0.01 ug/ml) and SEB (1 ug/ml). The control group was untreated. Cells were then aspirated out from three flasks after 2, 4, and 8 hour of incubations. They were washed with cold (2–8°C) 1X PBS, and centrifuged at 500 × g for 5 min at room temperature before labeled with Annexin V-FITC, PI, or a combination of Annexin V-FITC and PI (amounts of reagents added are according to the manufacture protocol, TACS Annexin V-FITC Apoptosis Detection Kit, R&D systems), respectively. Cells were analyzed in a Becton Dickinson FACSCaliber flow cytometer using Cell Quest software. At least 10,000 viable cells per condition were analyzed.

### Western blot analysis

Cell lines and cell culture media were purchased from ATCC (Manassas, VA). Cells were maintained in RPMI medium supplemented with 10% FBS and antibiotics. The Fas pathway was activated by the addition of 0.01 μg/ml of an anti-Fas activating antibody (clone DX2, BD Pharmingen, San Diego, CA) for normal cellular conditions and 1 μg/ml of SEB (Sigma Chemical, St. Louis, MO), in addition to the anti- Fas antibody, for perturbed conditions within the time range indicated (0–12 hours).

All blots were performed using standard procedures. In summary: following treatment, cells were lysed using M-PER lysis buffer (Pierce, Rockford IL) and the protein concentration was determined by BCA assay. A mass of 50 μg of total protein was loaded on a 10% polyacrylamide gel and separated by electrophoresis. Proteins were transferred to a nitrocellulose membrane and probed with specific antibodies (below) for 1 hour at room temperature. Blots were developed using ImmunoStar ECL reagent (BioRad, Hercules, CA) and imaged using a VersaDoc Imaging System (BioRad, Hercules, CA). Relative protein concentrations were determined using QuantityOne software (BioRad, Hercules, CA). Initial concentration values for the seven proteins assayed were simultaneously determined on the same nitrocellulose membrane.

The antibodies (Table 3) were obtained from Santa Cruz Biotechnology (Santa Cruz, CA) or R&D Systems (Minneapolis, MN) and used at 1 μg/ml.

### Relative protein level curves

Mean protein levels were calculated from a minimum of three independent replicates for each protein, ranging to nine replicates for some proteins. The five points for each protein curve (five time states: 0, 2, 4, 8, and 12 hours) were increased to 50 points with linear interpolation. Curve kurtosis was then reduced with a local polynomial regression fitting (loess smoothing). The 95% point-wise and simultaneous Bonferroni confidence intervals were calculated from the variance for each curve. All error terms and propagation of error given for time point intervals are calculated with the standard error of the mean.

### Ordinary differential equations (ODEs)

Initial value ODEs were determined from the model reactions (see Appendix) for each protein/protein complex. The equations were solved using a classical Runge-Kutta 4^th ^order integration function available in the 'odesolve' package of R [23]. The protein with the minimum initial level (active caspase-3) was used as the basal value for the protein complexes at (*t *= 0). This was determined to support the assumption that proteolytic activation of caspases (caspase-3 and caspase8) and protein complex formation occurs at minimum levels (thus at low concentrations) at initial time points.

### Optimization

The optimization problem seeks to minimize the aggregate Euclidean distance between the experimental and simulated data given as:

(1.1)da=∑j=1n(∑i=1t(xji−yji)2)12
 MathType@MTEF@5@5@+=feaafiart1ev1aaatCvAUfKttLearuWrP9MDH5MBPbIqV92AaeXatLxBI9gBaebbnrfifHhDYfgasaacH8akY=wiFfYdH8Gipec8Eeeu0xXdbba9frFj0=OqFfea0dXdd9vqai=hGuQ8kuc9pgc9s8qqaq=dirpe0xb9q8qiLsFr0=vr0=vr0dc8meaabaqaciaacaGaaeqabaqabeGadaaakeaafaqabeqacaaabaWaaeWaaeaacqaIXaqmcqGGUaGlcqaIXaqmaiaawIcacaGLPaaaaeaacqWGKbazdaWgaaWcbaGaemyyaegabeaakiabg2da9maaqahabaGaeiikaGYaaabCaeaacqGGOaakcqWG4baEdaWgaaWcbaGaemOAaOMaemyAaKgabeaakiabgkHiTiabdMha5naaBaaaleaacqWGQbGAcqWGPbqAaeqaaOGaeiykaKYaaWbaaSqabeaacqaIYaGmaaGccqGGPaqkdaahaaWcbeqaamaalmaameaacqaIXaqmaeaacqaIYaGmaaaaaaWcbaGaemyAaKMaeyypa0JaeGymaedabaGaemiDaqhaniabggHiLdaaleaacqWGQbGAcqGH9aqpcqaIXaqmaeaacqWGUbGBa0GaeyyeIuoaaaaaaa@5354@

where *x *and *y *are simulated and experimental protein concentrations, respectively, *n *is the total number of proteins, and *t *is the total number of times states. The 16 relative kinetic rate constants (parameters) were computed using a two-phase minimization approach over multiple trials. Due to the dependence between initial value seeding and the minimized function (local optima), a Monte Carlo simulation [24] was used to iteratively seed the initial parameter values to begin each optimization. The distance function was first minimized with a Newton-type non-linear procedure available in the 'stats' package of R [23]. The parameter values determined at convergence of the distance function were then seeded into a subsequent quasi-Newton minimization method with box constraints [25]. This method constrains the parameter values to a specified range in the minimization of the distance function. This final aggregate distance was stored in a list for each simulated trial.

**Figure 2:**
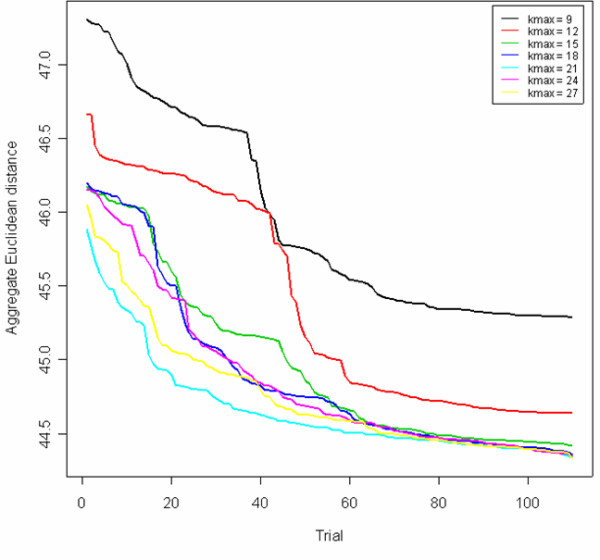
**Maximum rate constant range comparison**. Resulting minimum aggregate distances between experimental and simulated data for 150 initial value seedings (Monte Carlo simulation). Each curve represents the aggregated error (summated simulated vs. experimental) for the proteins in the model (y-axis) as a function of the trial (x-axis) using increasing maximum allowable relative rate constants. This allows the relative rate constants in the model to be constrained to the smallest range of values.

**Figure 3:**
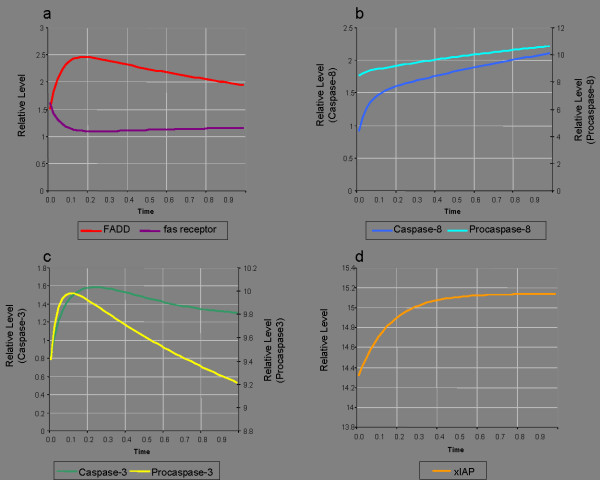
**Simulation results under normal cellularconditions**. Simulated protein profiles for the seven proteins assayed. (a) Fas receptor and FADD protein. (b) Inactive (procaspase-8) and active (capase-8) caspase-8 proteins. Note that the two proteins are plotted on the same graph with different scales. (c) Inactive (procaspase-3) and active (capase-3) caspase-3 proteins. Note that the two proteins are plotted on the same graph with different scales. (d) xIAP protein.

**Figure 4:**
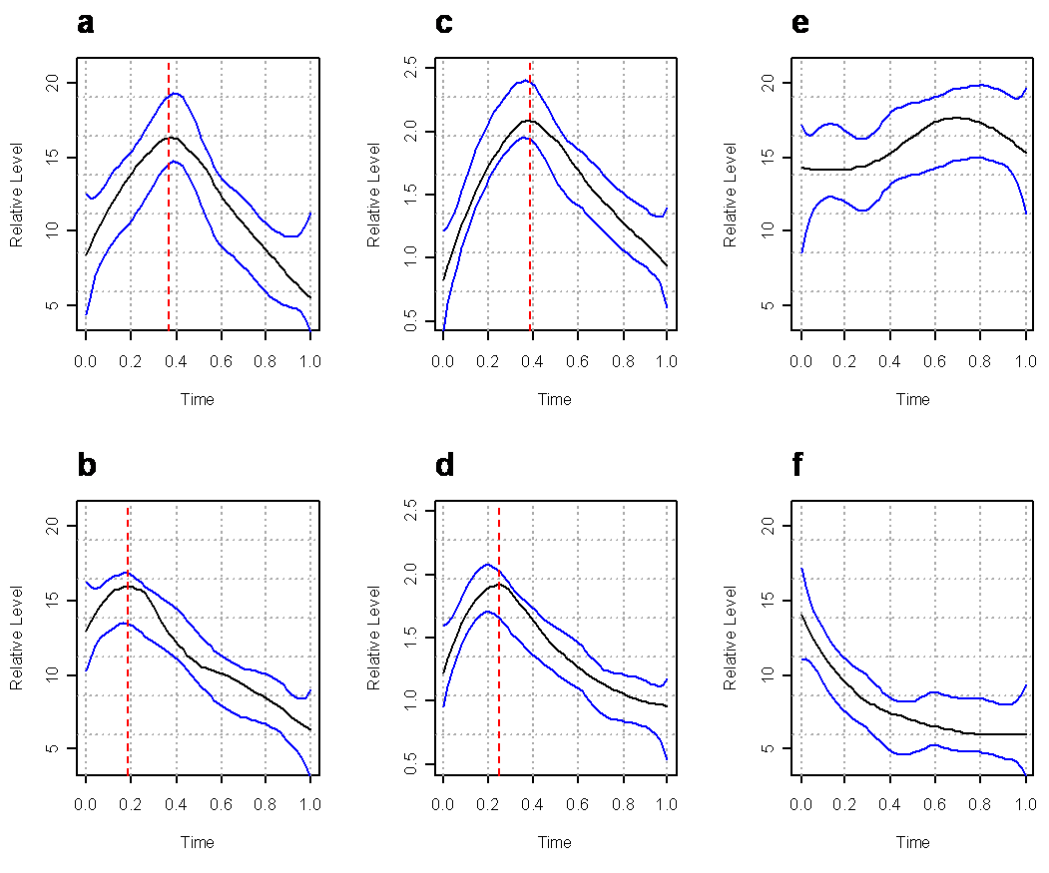
**Experimental data comparison between normal andSEB-exposed conditions**. (a) Procaspase-8 protein under normal cellular conditions. (b) Procaspase-8 protein under SEB-exposed conditions. (c) Caspase-3 protein under normal cellular conditions. (d) Caspase-3 protein under SEB-exposed conditions. (e) xIAP protein under normal cellular conditions. (f) xIAP protein under SEB-exposed conditions. Points in each plot are from a random sample from experimental values. Red dashed lines are placed at the time point where the peak occurs for each curve while blue lines represent 95% point-wise and simultaneous Bonferonni confidence bounds.

**Figure 5:**
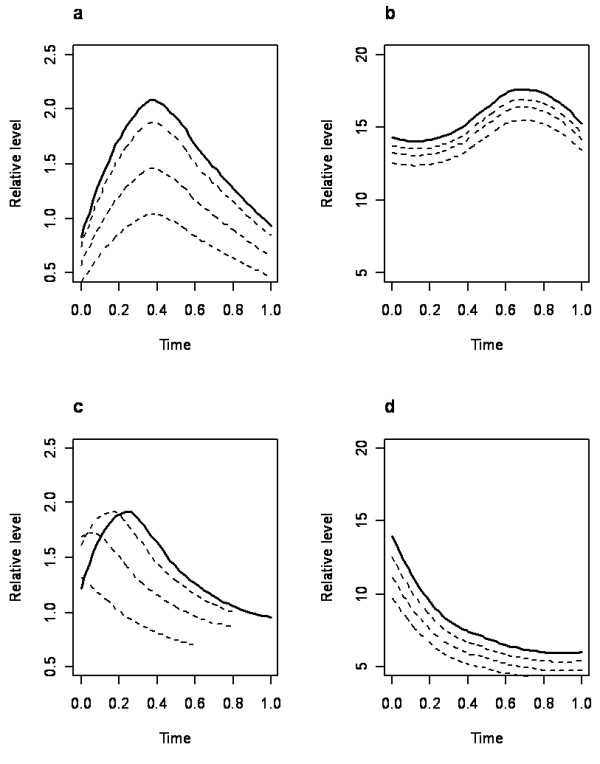
**Caspase-3 and xIAP under normal and simulated SEBconditions**. (a) Caspase-3 potential solutions where a peak in expression occurs at 4.65 ± 0.18 hours. (b) xIAP potential solutions where the protein is either constitutively expressed or simply positively expressed after *t *= 0. (c) Simulated potential solutions for caspase-3, where the rate of synthesis is increased from 4.65 hours. Note that the level of expression can fluctuate with an increased rate. (d) Simulated potential solutions for xIAP, where the protein has negative expression levels after *t *= 0. The multiple dashed lines for each plot represent the various degrees of potential protein levels that can occur, while maintaining the expected pattern.

**Figure 6:**
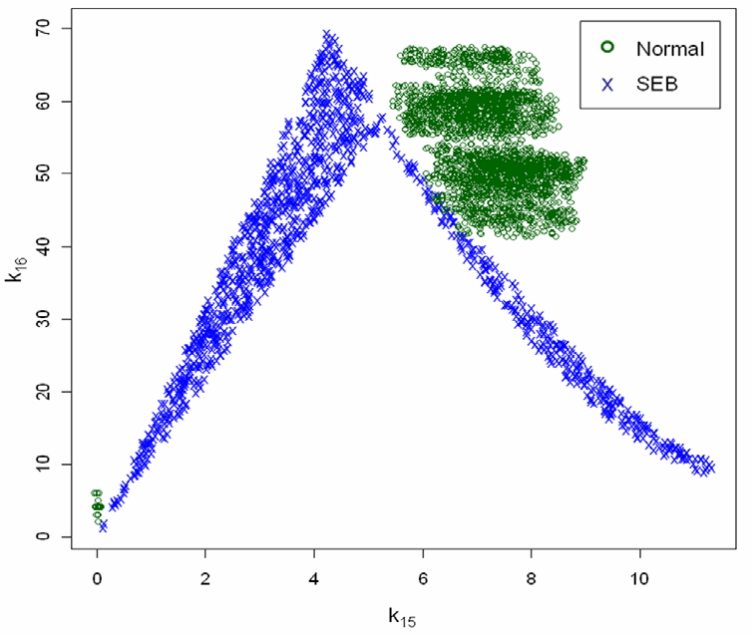
**Rate space for caspase-3 and xIAP synthesis forsingle regulatory reaction**. Plot of simulated solutions to normal conditions and SEB-exposed conditions within varied values of relative rate constants k_16 _vs. k_15_. Normally distributed random noise (jitter) has been added to the green points to reduce the overlap and illustrate the frequency of simulations.

For each iteration in the Monte Carlo simulation, the parameter values were sampled from a random uniform distribution with a lower bound near zero and an upper bound of *λ*. The *λ *variable was increased from an initial value of nine to a maximum value of *λ *= 27. This procedure was implemented for determination of the minimum allowable parameter value range with the minimum distance function. Such a method constrains the rate constants to a small range of values. The results are represented in Figure 2. Each line corresponds to a different maximum parameter value *λ *over a series of 150 minimization trials. When the 16 parameter values are confined to the range of near-zero (1.0 × 10^-9^) to *λ *= 9, the minimum aggregate distance obtained over 150 trials is 45.48. However, as the maximum allowable parameter value is increased to *λ *= 18, the minimum aggregate distance is reduced to 44.46. Additionally, all maximum parameter values *λ *≥ 18 converge to the same minimum aggregate distance. This result indicates that the distance function is not reduced any further with a maximum parameter value greater than *λ *= 18. As such, the range of rate constants was limited to values of *λ *= 1.0 × 10^-9 ^to *λ *= 18.

### Hotelling's two-sample *T*^2 ^test

For assignment of a statistical summary to the difference between each protein under normal and SEB-exposed conditions, a *T*^2 ^test was calculated, using all replicates for each protein. This multivariate analog to the Student's *t*-test is implemented to account for the correlation structure between the *t *time state variables. The squared distance is given by:

(2.1)     *T*^2 ^= *n *(X¯
 MathType@MTEF@5@5@+=feaafiart1ev1aaatCvAUfKttLearuWrP9MDH5MBPbIqV92AaeXatLxBI9gBaebbnrfifHhDYfgasaacH8akY=wiFfYdH8Gipec8Eeeu0xXdbba9frFj0=OqFfea0dXdd9vqai=hGuQ8kuc9pgc9s8qqaq=dirpe0xb9q8qiLsFr0=vr0=vr0dc8meaabaqaciaacaGaaeqabaqabeGadaaakeaacuWGybawgaqeaaaa@2DFD@- *μ*_0_)^T ^(*S*)^-1 ^(X¯
 MathType@MTEF@5@5@+=feaafiart1ev1aaatCvAUfKttLearuWrP9MDH5MBPbIqV92AaeXatLxBI9gBaebbnrfifHhDYfgasaacH8akY=wiFfYdH8Gipec8Eeeu0xXdbba9frFj0=OqFfea0dXdd9vqai=hGuQ8kuc9pgc9s8qqaq=dirpe0xb9q8qiLsFr0=vr0=vr0dc8meaabaqaciaacaGaaeqabaqabeGadaaakeaacuWGybawgaqeaaaa@2DFD@ - *μ*_0_), where

(2.2)X¯(t×1)=1n∑j=1nXi
 MathType@MTEF@5@5@+=feaafiart1ev1aaatCvAUfKttLearuWrP9MDH5MBPbIqV92AaeXatLxBI9gBaebbnrfifHhDYfgasaacH8akY=wiFfYdH8Gipec8Eeeu0xXdbba9frFj0=OqFfea0dXdd9vqai=hGuQ8kuc9pgc9s8qqaq=dirpe0xb9q8qiLsFr0=vr0=vr0dc8meaabaqaciaacaGaaeqabaqabeGadaaakeaafaqabeqacaaabaWaaeWaaeaacqaIYaGmcqGGUaGlcqaIYaGmaiaawIcacaGLPaaaaeaacuWGybawgaqeamaaBaaaleaacqGGOaakcqWG0baDcqGHxdaTcqaIXaqmcqGGPaqkaeqaaaaakiabg2da9maalaaabaGaeGymaedabaGaemOBa4gaamaaqahabaGaemiwaG1aaSbaaSqaaiabdMgaPbqabaaabaGaemOAaOMaeyypa0JaeGymaedabaGaemOBa4ganiabggHiLdaaaa@45D5@, and

(2.3)S(t×t)=1n−1∑j=1n(Xi−X¯)(Xi−X¯)T
 MathType@MTEF@5@5@+=feaafiart1ev1aaatCvAUfKttLearuWrP9MDH5MBPbIqV92AaeXatLxBI9gBaebbnrfifHhDYfgasaacH8akY=wiFfYdH8Gipec8Eeeu0xXdbba9frFj0=OqFfea0dXdd9vqai=hGuQ8kuc9pgc9s8qqaq=dirpe0xb9q8qiLsFr0=vr0=vr0dc8meaabaqaciaacaGaaeqabaqabeGadaaakeaafaqabeqacaaabaWaaeWaaeaacqaIYaGmcqGGUaGlcqaIZaWmaiaawIcacaGLPaaaaeaacqWGtbWudaWgaaWcbaGaeiikaGIaemiDaqNaey41aqRaemiDaqNaeiykaKcabeaaaaGccqGH9aqpdaWcaaqaaiabigdaXaqaaiabd6gaUjabgkHiTiabigdaXaaadaaeWbqaaiabcIcaOiabdIfaynaaBaaaleaacqWGPbqAaeqaaOGaeyOeI0IafmiwaGLbaebacqGGPaqkcqGGOaakcqWGybawdaWgaaWcbaGaemyAaKgabeaakiabgkHiTiqbdIfayzaaraGaeiykaKYaaWbaaSqabeaacqqGubavaaaabaGaemOAaOMaeyypa0JaeGymaedabaGaemOBa4ganiabggHiLdaaaa@5423@ and

(2.4)μ0(t×1)=[μ10μ20..μt0]
 MathType@MTEF@5@5@+=feaafiart1ev1aaatCvAUfKttLearuWrP9MDH5MBPbIqV92AaeXatLxBI9gBaebbnrfifHhDYfgasaacH8akY=wiFfYdH8Gipec8Eeeu0xXdbba9frFj0=OqFfea0dXdd9vqai=hGuQ8kuc9pgc9s8qqaq=dirpe0xb9q8qiLsFr0=vr0=vr0dc8meaabaqaciaacaGaaeqabaqabeGadaaakeaafaqaaeqacaaabaWaaeWaaeaacqaIYaGmcqGGUaGlcqaI0aanaiaawIcacaGLPaaaaeaaiiGacqWF8oqBdaWgaaWcbaGaeGimaaZaaSbaaWqaaiabcIcaOiabdsha0jabgEna0kabigdaXiabcMcaPaqabaaaleqaaOGaeyypa0ZaamWaaeaafaqaaeqbbaaaaeaacqWF8oqBdaWgaaWcbaGaeGymaeJaeGimaadabeaaaOqaaiab=X7aTnaaBaaaleaacqaIYaGmcqaIWaamaeqaaaGcbaGaeiOla4cabaGaeiOla4cabaGae8hVd02aaSbaaSqaaiabdsha0jabicdaWaqabaaaaaGccaGLBbGaayzxaaaaaaaa@4AF4@

such that at large values of *T*^2^, the hypothesis *H*_0_: *μ *= *μ*_0 _is rejected. The *T*^2 ^statistic is distributed as (n−1)t(n−t)
 MathType@MTEF@5@5@+=feaafiart1ev1aaatCvAUfKttLearuWrP9MDH5MBPbIqV92AaeXatLxBI9gBaebbnrfifHhDYfgasaacH8akY=wiFfYdH8Gipec8Eeeu0xXdbba9frFj0=OqFfea0dXdd9vqai=hGuQ8kuc9pgc9s8qqaq=dirpe0xb9q8qiLsFr0=vr0=vr0dc8meaabaqaciaacaGaaeqabaqabeGadaaakeaadaWcaaqaamaabmaabaGaemOBa4MaeyOeI0IaeGymaedacaGLOaGaayzkaaGaemiDaqhabaWaaeWaaeaacqWGUbGBcqGHsislcqWG0baDaiaawIcacaGLPaaaaaaaaa@3844@*F*_*t*,*n*-*t*_, where *F*_*t*,*n*-*t*_, is a random variable that follows an *F *– distribution with *t *and *n *- *t *degrees of freedom [26]. For each comparison, Bartlett's test was computed to verify the homogeneity of the covariance matrices

## Authors' contributions

The modeling and simulation was conducted primarily by BWH and WEC. The experimental results were obtained by HBS and JD. BWH, JD, WEC, and HBS drafted the manuscript. The critical assessment of results and scientific discussions were contributed by OJP, JCF, RH, and MJ. All authors read and approved the final manuscript.

## Supplementary Material

Additional File 1 **Model equations**. Series of 13 initial value ODEs used for the model of Fas-mediated apoptosis. Click here for file

## Figures and Tables

**Table 1:** Correlation between experimental and simulated data The p-values are calculated for a one-sided alternative hypothesis, assuming a positive association between the experimental and simulated curves for each protein (H_0_: β= 0 vs. H_1_: β > 0).

Protein	Pearson's correlation coefficient	p-value
Fas receptor	0.70	< 0.0001
FADD	0.83	< 0.0001
Procaspase-8	-0.48	0.9998
Procaspase-3	0.97	< 0.0001
Caspase-8	0.95	< 0.0001
Caspase-3	0.81	< 0.0001
xIAP	0.71	< 0.0001

**Table 2:** Summary of Hotelling's *T*^2 ^two-sample test. For a size α = 0.05 test, there is a significant (denoted with a '*' symbol) difference between normal and SEB-exposed conditions for procaspase-8, caspase-3, and xIAP.

Protein	*T*^2 ^value	*F*-value	df (*t*, *n*-*t*)	p-value
Fas receptor	2.73	0.48	4,7	0.75
FADD	33.48	4.78	4,4	0.079
Procaspase-8	101.32	14.47	4,4	0.012*
Procaspase-3	8.00	1.14	4,4	0.45
Caspase-8	33.51	4.79	4,4	0.079
Caspase-3	54.60	9.56	4,7	0.006*
xIAP	113.96	16.28	4,4	0.01*

**Table 3:** Antibody information

**Protein**	**Clone**	**Species**	**Supplier**	**Note**
Caspase-3	N-19	Goat	Santa Cruz	Precursor form
Caspase-3	269518	Rabbit	R&D Systems	Active form
Caspase-8	S-19	Goat	Santa Cruz	Precursor form
Caspase-8	AF832	Rabbit	R&D Systems	Active form
xIAP	48	Mouse	BD Pharmingen	
FADD	S-18	Goat	Santa Cruz	
Fas	B-10	Goat	Santa Cruz	
