# Supplementary material for: Modeling the effects of a Staphylococcal Enterotoxin B (SEB) on the apoptosis pathway
Source: BMC Microbiol. 2006 May 31;6:48. doi: 10.1186/1471-2180-6-48 (PMC1489937; doi:10.1186/1471-2180-6-48)
Supplement: Additional File 1 — Model equations. Series of 13 initial value ODEs used for the model of Fas-mediated apoptosis. [file 1471-2180-6-48-S1.pdf]

Model equations

d[FasR]/*dt*=(k_2[Fas.Rec.Lig])-(k_1[FasR][FasL])

d[FasL]/*dt*=(k_2[Fas.Rec.Lig])-(k_1[FasR][FasL])

d[Fas.Rec.Lig]/*dt*=(k_1[FasR][FasL]+k_4[FasComplex])-(k_2[Fas.Rec.Lig]+k_3[Fas.Rec.Lig][FADD])

d[FADD]/*dt*=(k_4[FasComplex]+k_9[P8.FasComplex]-(k_3[Fas.Rec.Lig][FADD]+k_{10}[Procaspase3][Caspase3][FADD])

d[FasComplex]/*dt*=(k_3[Fas.Rec.Lig][FADD]+k_6[P8.FasComplex])-(k_4[FasComplex]+k_5[Procaspase8][FasComplex][Procaspase3]+k_8[Caspase8][FasComplex][Procaspase8])

d[P8.FasComplex]/*dt*=(k_5[Procaspase8][FasComplex][Procaspase3]+k_8[Caspase8][Procaspase8]+k_{10}[Procaspase3][Caspase3][FADD])-(k_6[P8.FasComplex]+k_7[P8.FasComplex]+k_9[P8.FasComplex])

d[Procaspase8]/*dt*=(k_6[P8.FasComplex]+k_7[P8.FasComplex])-(k_5[Procaspase8][FasComplex][Procaspase3]+k_8[Caspase8][Procaspase8])

d[Procaspase3]/*dt*=(k_{12}[C8.P3.Complex]+k_{13}[C8.P3.Complex]+k_6[P8.FasComplex]+k_9[P8.FasComplex])-(k_{11}[Caspase8][Procaspase3]+k_{10}[Procaspase3][Caspase3][FADD]+k_{14}[Caspase8][Caspase3][Procaspase3]+k_5[Procaspase8][FasComplex][Procaspase3])

d[Caspase8]/*dt*=(k_7[P8.FasComplex]+k_{12}[C8.P3.Complex]+k_{13}[C8.P3.Complex])-(k_{11}[Caspase8][Procaspase3]+k_8[Caspase8][Procaspase8]+k_{14}[Caspase8][Caspase3][Procaspase3])

d[Caspase3]/*dt*=(k_{13}[C8.P3.Complex]+k_{16}[inhi.Complex3]+k_9[P8.FasComplex])-(k_{15}[Caspase3][CIAP]+k_{14}[Caspase8][Caspase3][Procaspase3]+k_{10}[Procaspase3][Caspase3][FADD])

d[C8.P3.Complex]/*dt*=(k_{11}[Caspase8][Procaspase3]+k_{14}[Caspase8][Caspase3][Procaspase3])-(k_{12}[C8.P3.Complex]+k_{13}[C8.P3.Complex])

d[xIAP]/*dt*=(k_{16}[inhi.Complex3])-(k_{15}[Caspase3][xIAP])

d[inhi.Complex3]/*dt*=(k_{15}[Caspase3][xIAP])-(k_{16}[inhi.Complex3])
